# Arsenical Treatment of Chorea

**Published:** 1882-07

**Authors:** 


### ARSENICAL TREATMENT OF CHOREA.

M. Siredey, we read in the *Revue de Thèrap. Méd. et Chirurg.*, has long had recourse to arsenic in the treatment of rheumatismal chorea, and has had good results from this medication. He uses the solution of Bondin :

℞ Ac. arsenious......................................gr. xv.
Aquæ destill.......................................Oij. M.

This is allowed to boil for about a quarter of an hour, and is then ready for use. The extreme dilution of the solution is favorable for its use among children. For a child from six to ten years of age, about one drachm and a half may be given daily, in divided doses, in sweetened water. In this way the medicament is generally well tolerated.

Dr. Sottmann, of the Breslau *Clinic*, constantly prescribes the following solution in chorea :

℞ Liquor Fowleri.................................m iv.
Aquæ.................................................3 ij. M

This quantity to be taken in divided doses daily. He generally obtains a complete cure in from sixteen to twenty-one days.

In some cases he adds from eight to fifteen grains of chloral to the prescription. He has generally found that anæmic children, and those of hereditary nervous dispositions, bear this form of medication well.—*Medical and Surgical Reporter.*

